# Tuberculous Arthritis of the Elbow Joint: An Uncommon Location with a Diagnostic Dilemma

**DOI:** 10.7759/cureus.2462

**Published:** 2018-04-11

**Authors:** Neelam Khetpal, Sameen Khalid, Ranjeet Kumar, Manuel F Betancourt, Akash Khetpal, Christopher Wasyliw, Seema Patel

**Affiliations:** 1 Internal Medicine Residency, Florida Hospital-Orlando, Orlando, USA; 2 Critical Care Medicine, Florida Hospital-Orlando, Orlando, USA; 3 DMC, Dow University of Health Sciences (DUHS), Karachi, Pakistan; 4 Diagnostic Radiology, Florida Hospital-Orlando, Orlando, USA; 5 Infectious Diseases, Florida Hospital-Orlando, Orlando, USA

**Keywords:** tuberculosis, arthritis

## Abstract

Musculoskeletal tuberculosis accounts for 1%-3% of all cases of tuberculosis (TB) worldwide with elbow involvement being even less common. The most cases of tuberculous arthritis occur in patients born in and emigrated from endemic regions, especially in patients who are co-infected with human immunodeficiency virus (HIV). We present a rare case of tuberculous septic arthritis of the elbow joint in a 78-year-old African-American female from the United States, with no history of travel abroad. Her presenting symptoms included pain, swelling, and decreased range of motion of the right elbow for six months. She underwent incision and debridement of the elbow joint and was started on empiric intravenous antibiotic therapy for suspected pyogenic septic arthritis. Several weeks later, surgical cultures demonstrated acid-fast bacilli, identified as Mycobacterium tuberculosis (M. tuberculosis) and a four-drug anti-tuberculosis regimen was initiated. Based upon culture results, additional imaging evaluation was undertaken. She did not have any symptoms of a pulmonary disease but was found to be positive for Mycobacterium tuberculosis in sputum cultures and bronchoalveolar lavage. We emphasize the importance of considering a tuberculosis infection in the differential diagnosis of monoarticular arthritis, especially in elderly patients with immune deficient states since early recognition and treatment result in good functional outcomes.

## Introduction

Infections due to Mycobacterium tuberculosis (M. tuberculosis) occur worldwide, with humans being the only known reservoir. Of the estimated two to three billion people infected with M. tuberculosis, only a relatively small percentage (5%-15%) of people will develop clinical disease [1]. Lungs are the most common organ of involvement. Extrapulmonary tuberculosis (TB) makes up about 15%-20% of all cases of TB in immunocompetent hosts and 50% in those individuals who are affected with human immunodeficiency virus (HIV) [2]. Musculoskeletal TB accounts for 35% of extrapulmonary TB cases [3] and 1%-3% of all TB cases worldwide [4-5]. The most commonly infected joints are the spine, hip, and knee in order of frequency [5]. An infection of the elbow joint is particularly rare, making up 1%-5% of all cases of musculoskeletal TB [6]. We are presenting a rare case of a 78-year-old female patient who presented with a presumptive diagnosis of pyogenic septic arthritis, which was later unmasked as tuberculous septic arthritis of the elbow.

## Case presentation

A 78-year-old African-American female presented to the hospital with complaints of worsening right elbow pain and swelling for six months. She described the pain as a dull ache that worsened with movement and, over time, was unable to fully move the arm. She denied any history of trauma or any other joint involvement. She had a past medical history of stage IIIC ovarian Mullerian tumor with peritoneal carcinomatosis, requiring abdominal hysterectomy with bilateral salpingo-oophorectomy and neoadjuvant chemotherapy with paclitaxel and carboplatin, which was completed two years ago. She was born in the United States and was a retired accountant. She denied any travel outside the country or exposure to TB cases in the United States. Her medications included meloxicam and alendronate.

**Figure 1:**
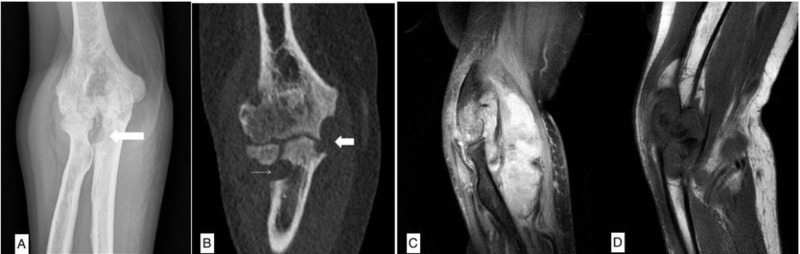
(1A) Radiograph of the right elbow demonstrating significant diffuse joint space narrowing and a periarticular erosion in the radial notch of the ulna (arrow). (1B) Computed tomography (CT) of the right elbow showing periarticular erosions medially at the elbow, involving the humeral trochlea and ulnar olecranon (left arrow) and an erosion in the radial notch of the ulna (right arrow). (1C) Sagittal T2 fat-saturated magnetic resonance imaging (MRI) of the right elbow, illustrating a large effusion with a heterogeneous signal consistent with a synovitis. (1D) Sagittal T1 MRI of the right elbow revealing a large erosion in the ulnar olecranon and distal humerus with the replacement and infiltration of the normal marrow signal consistent with osteomyelitis

On initial presentation, her vitals were significant for a temperature of 102.6 F, a respiratory rate of 22 breaths/minute, a pulse rate of 87/minute, and blood pressure of 124/66 mm Hg. The physical examination was remarkable for right elbow erythema, swelling, effusion, and tenderness to palpation. Her range of motion was restricted to only five degrees of flexion and extension at the right elbow joint. The laboratory evaluation revealed leukocytosis of 12.3 x 103/µL, a uric acid level of 7.1 mg/dL, a C-reactive protein of 115 mg/L, and an erythrocyte sedimentation rate of 104 mm/h. An X-ray and computed tomography (CT) of the elbow joint demonstrated bony erosive changes, joint space narrowing, and large effusion in the elbow joint. Magnetic resonance imaging (MRI) of the elbow joint revealed large joint effusion, synovitis, and osteomyelitis of the surrounding bones (Figure 1). Blood cultures were drawn and the patient was started on intravenous vancomycin and piperacillin-tazobactam for presumptive pyogenic septic arthritis. She underwent incision and drainage of the right elbow after an unsuccessful attempt at arthrocentesis. Preliminary blood and surgical cultures were negative for bacterial growth. She was discharged home with empiric intravenous antibiotic therapy for six weeks, home wound care, and occupational therapy.

**Figure 2:**
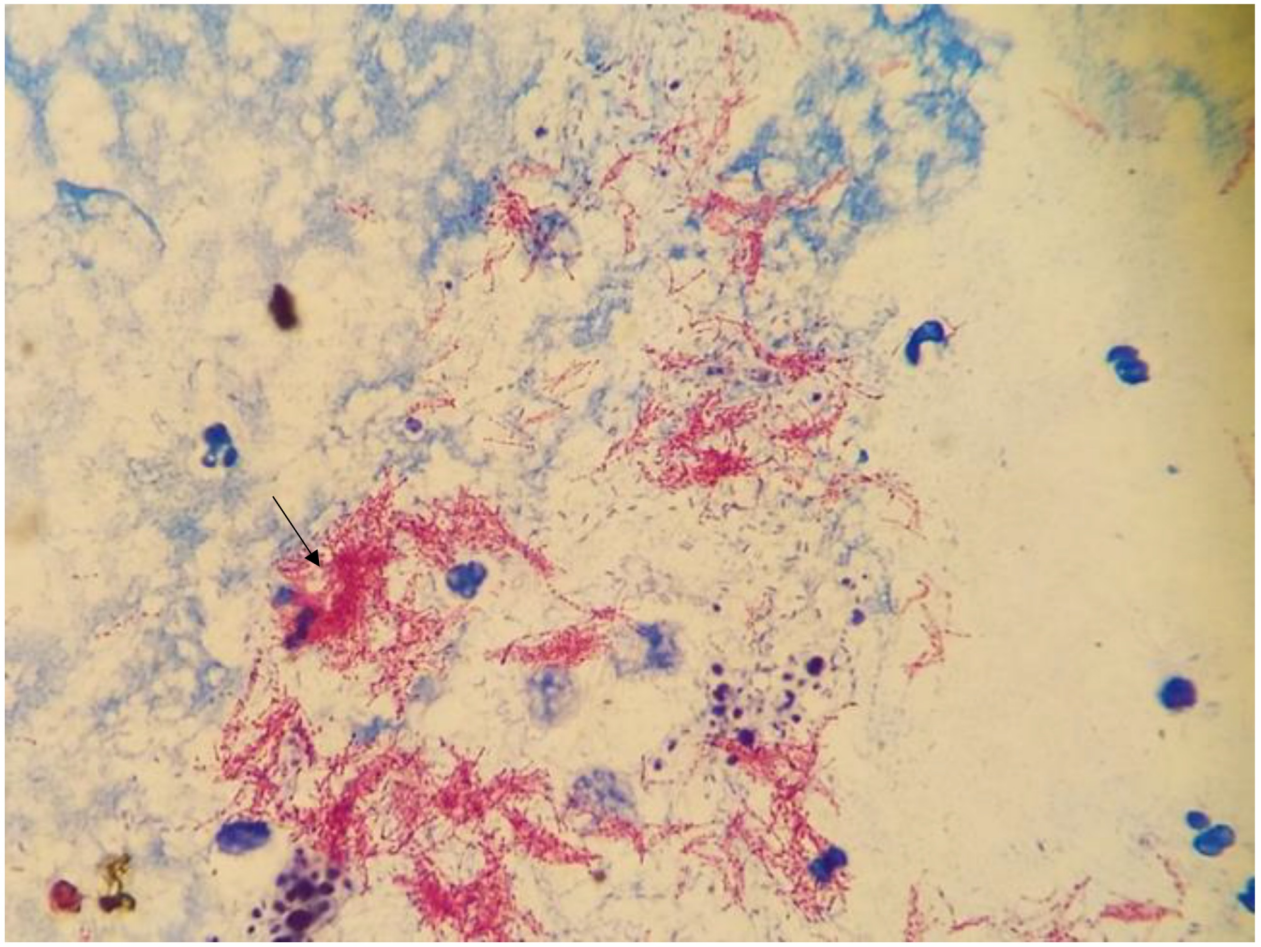
Carbolfuchsin stain from a direct specimen of the right elbow joint showing acid-fast bacilli (arrow)

**Figure 3:**
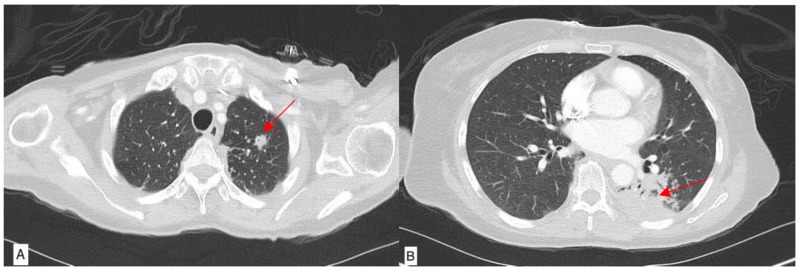
Computed tomography (CT) of the chest showing (3A) pulmonary nodule at the left lung apex (arrow), (3B) dense area of consolidation in the left lower lobe (arrow)

The patient’s symptoms initially improved but about three weeks later, she returned to the hospital with worsening right elbow pain and swelling. Acid-fast bacillus (AFB) cultures sent at the time of initial incision and drainage became positive for acid-fast bacilli (Figure 2), and her antibiotics were switched to azithromycin, meropenem, and doxycycline to cover for atypical mycobacteria. A deoxyribonucleic acid (DNA) probe of the acid-fast bacilli was positive for Mycobacterium tuberculosis (MTB), and the patient was started on four-drug anti-tuberculosis therapy, including rifampin, ethambutol, isoniazid, and pyrazinamide with pyridoxine. Final culture results confirmed the presence of the Mycobacterium tuberculosis complex and susceptibilities revealed a non-resistant MTB strain. She was then evaluated for disseminated disease with a CT chest, abdomen, and pelvis. Her CT chest results were remarkable for multiple left, upper lobe nodules and left lower lobe air-space consolidation (Figure 3). The patient denied ever having any symptoms of cough, shortness of breath, hemoptysis, and weight loss. She did report having night sweats and chronic fatigue. Bronchoscopy was performed and bronchoalveolar lavage fluid cultures were positive for MTB. She was diagnosed with active TB of the lungs and the elbow joint, possibly as a reactivation of latent TB. The HIV test was negative. The patient was discharged home on the four-drug anti-tuberculous regimen for 12 months under the supervision of the health department. At a four-month follow-up visit, the patient showed significant improvement in her symptoms.

## Discussion

An estimated 2-3 billion persons worldwide have latent TB infection and only 5%-15 % develop an active infection during their lifetime [1]. Lungs are the most common site of involvement with active TB. About 25% have asymptomatic pulmonary infection [7]. Over the past six decades, TB case rates have been consistently trending down; however, susceptible groups remain at high risk in the United States (U.S.) African-Americans represent the highest racial group, constituting 37% of TB cases among the U.S.-born persons [8]. Predisposing factors for TB in the United States include birth in TB endemic areas and recent immigration, poor socioeconomic conditions, injection and non-injection substance abuse, diabetes, hematological malignancy, head and neck solid organ malignancy, aging, and immunodeficiency, including HIV, solid organ transplant, and tumor necrosis factor-alpha inhibitors [2,9-10].

Musculoskeletal involvement occurs from a dormant pulmonary or extra-osseous focus by a hematogenous, lymphatic, or direct spread of bacilli [6]. Tuberculosis of the elbow usually starts with olecranon or the lower end of the humerus, but in some cases, the primary lesion involves the synovium or the upper end of the radius [11]. The tuberculous lesion is almost always a combination of osteomyelitis and arthritis. Initially, an inflammatory reaction develops in the synovium followed by granulation tissue formation, progressing to the development of effusion with fibrin deposition, forming “rice” bodies. This pannus of granulation tissue then begins to destroy cartilage, leading to bone demineralization and caseous necrosis. Cartilage is destroyed peripherally, preserving joint space for a considerable period of time, which has important clinical implications [12].

Tuberculous arthritis usually presents insidiously with chronic joint pain accompanied by swelling, progressive loss of function, and local muscle wasting. Constitutional symptoms, including low-grade fever, weight loss, lassitude, night sweats, anemia, and tachycardia, are present in a significant minority of cases [4]. In approximately 50% of the cases, symptoms and radiographic evidence of pulmonary TB are absent and tuberculosis is frequently missed as a differential diagnosis of the chronic inflammation of joints in the absence of active pulmonary disease. The differential diagnoses of skeletal TB include pyogenic arthritis, rheumatoid arthritis, sarcoid arthritis, gout, pigmented villonodular synovitis, and tumors [6].

Early diagnosis of skeletal TB is essential since the preservation of joint space early in the disease leads to good functional outcomes if the condition is treated in the early stages. A tuberculin skin test, usually positive in musculoskeletal TB, is one of the most valuable diagnostic tests [12]. Confirmation by microscopy and culture is necessary, and the joint fluid aspirate should be evaluated for smear and culture, as it yields positive culture results in 80% of the cases [3,12]. A clear synovial aspirate is an excellent specimen for the polymerase chain reaction and nucleic acid probes [4]. On a plain radiograph, a Phemister triad suggesting tuberculosis arthritis may be observed in some cases. This triad includes juxta-articular osteopenia, peripherally located osseous erosions, and a gradual narrowing of the joint space [5]. Martini et al. divided the radiological presentation of osteoarticular tuberculosis into four stages: stage 1 - localized osteoporosis, but no bony lesion; stage 2 - one or more erosions or cavities in bones; stage 3 - involvement of the whole joint without gross destruction; stage 4 - gross destruction [13]. MRI and CT scans can further aid in diagnosis, especially in determining the extent of the disease. A definitive diagnosis can be established by obtaining a biopsy specimen from diseased tissue, synovium, or bone and demonstrating positive cultures for Mycobacterium tuberculosis and caseating granulomas [3,4,6].

The cornerstone of treatment for musculoskeletal TB is three to four drug regimens based on sensitivity results. The optimal duration of treatment is uncertain with the conventional duration being 12-18 months. However, recent data suggest that the duration of six to nine months of therapy can be appropriate in regimens containing rifampin [2]. Good functional recovery can be obtained with prompt diagnosis and treatment with anti-tuberculous chemotherapy and early mobilization. Surgery is rarely necessary in the modern era since the advent of chemotherapy. Advanced cases of peripheral joint involvement can be successfully treated with synovectomy, debridement of necrotic tissue, and abscess drainage without needing arthrodesis. With effective chemotherapy, surgery should be reserved only to prevent deformities and improve function when the disease has not responded to chemotherapy well [12].

## Conclusions

Tuberculosis of the elbow joint, in particular, is a rare entity and very few cases have been reported in the United States. Our case is unique because the patient did not have any exposure or significant risk factors for TB. Her presentation of refractory elbow pain led to further workup and an eventual diagnosis of tuberculous arthritis of the elbow as well as concurrent pulmonary tuberculosis, which was otherwise asymptomatic. Our case serves as a reminder to consider osteoarticular tuberculosis in the differential diagnosis of chronic monoarticular joint pain and swelling even if significant risk factors are absent. We stress the importance of early diagnosis and treatment to prevent the rapid progression of the disease and poor functional recovery at late stages.
